# Physical activity, sedentary behaviors and all-cause mortality in patients with heart failure: Findings from the NHANES 2007–2014

**DOI:** 10.1371/journal.pone.0271238

**Published:** 2022-07-15

**Authors:** Youngdeok Kim, Justin M. Canada, Jonathan Kenyon, Hayley Billingsley, Ross Arena, Carl J. Lavie, Salvatore Carbone

**Affiliations:** 1 Department of Kinesiology & Health Sciences, College of Humanities & Sciences, Virginia Commonwealth University, Richmond, VA, United States of America; 2 Division of Cardiology, Department of Internal Medicine, VCU Pauley Heart Center, Virginia Commonwealth University, Richmond, VA, United States of America; 3 Department of Physical Therapy, College of Applied Health Sciences, University of Illinois at Chicago, Chicago, IL, United States of America; 4 John Ochsner Heart and Vascular Institute, Ochsner Clinical School-the University of Queensland School of Medicine, New Orleans, LA, United States of America; UT Southwestern: The University of Texas Southwestern Medical Center, UNITED STATES

## Abstract

**Background:**

Limited data are available examining the effects of both moderate- and vigorous-intensity physical activity (MVPA) and sedentary behavior (SB) on longevity among patients with heart failure (HF). This study examined the associations of MVPA and SB with all-cause mortality in HF patients using a nationally representative survey data.

**Methods:**

National Health and Nutrition Examination Survey data (2007–2014) were used. 711 adults with self-reported congestive HF, linked to 2015 mortality data were analyzed. Self-reported MVPA and SB minutes were used to create the three MVPA [No-MVPA, insufficient (I-MVPA; <150 min/wk), and sufficient (S-MVPA; ≥150 min/wk)] and two SB (<8 and ≥8 hrs/d) groups. Cox proportional hazard models were constructed to test the associations of MVPA and SB with all-cause mortality.

**Results:**

119 deaths occurred over an average of 4.9 years of follow-up. Lower MVPA and higher SB were independently associated with poor survival (*P* < .001). Joint and stratified analyses showed that the protective effect of MVPA was most pronounced among patients with SB<8 hrs/d. There was no difference in the mortality risk by SB levels within I-MVPA and S-MVPA groups; however, in the No-MVPA group, those with SB≥8 hrs/d had a greater risk of mortality compared to those with <8 hrs/d (Hazard ratio = 1.60).

**Conclusion:**

In this HF cohort, MVPA and SB were independently and jointly associated with all-cause mortality. The beneficial effect of MVPA is attenuated by excessive SB; however, engaging in some amount of MVPA may provide a protective effect and attenuates the detrimental effects associated with excessive SB.

## Introduction

The clinical syndrome of heart failure (HF) affects more than 6 million adults in the United States (US) [1] and its prevalence is projected to increase by 46% between 2012 and 2030 [2]. Importantly, HF remains the leading cause of hospitalizations in older adults and, despite numerous advances in understanding its pathophysiology and related targeted therapeutic strategies, mortality remains unacceptably high [3]. This highlights the continued need to assess therapeutic strategies that can slow the progression of HF as well as improve common clinical symptoms caused by HF (e.g., exertional dyspnea and fatigue with daily activities).

Physical activity (PA) is an essential lifestyle behavior associated with better cardiovascular (CV) health. A large body of literature supports that adherence to current PA guidelines, including ≥150 minutes of moderate- to vigorous-intensity PA (MVPA), is strongly associated with a reduced risk of CV disease (CVD)-related morbidity and mortality [4]. More recently, excessive sedentary behavior (SB), which is defined as any waking behaviors performed at low energy expenditure (<1.5 metabolic equivalents [METs]) while in seated or reclined postures, has also been recognized as a potential lifestyle risk factor negatively associated with human health, including the development of HF among the general population [5].

Although an increased level of PA and reduced SB have been associated with a reduced risk for HF [6], there is a paucity of evidence related to the effects of PA and SB on survival in patients with established HF. Moreover, in individuals without HF, it has been proposed that the detrimental effects of SB may be attenuated or even eliminated by increasing levels of MVPA [7, 8]; however, such joint effects have not been studied among patients with established HF.

The primary objectives of this study were to: 1) examine the independent effects of MVPA and SB on all-cause mortality in patients with HF; and 2) investigate the potential joint and interactive effects of MVPA and SB using combined and stratified analyses. Additionally, we examined the prevalence and demographic correlates of MVPA and SB to better identify the characteristics of the adults with HF that may influence MVPA and SB behaviors.

## Methods

### Study population

We used the data from the National Health and Nutrition Examination Survey (NHANES) conducted by the Centers for Disease Control and Prevention (CDC), which is an ongoing, cross-sectional survey designed to monitor the various aspects of the health of the US population in two-year cycles. The NHANES selects the participants using a complex, multistage probability sampling method producing a nationally representative sample of the noninstitutionalized US population. Participants complete a set of questionnaires during a household interview conducted by a trained interviewer followed by a standardized physical examination in mobile examination centers. The study protocols of the NHANES are approved by the Research Ethics Review Board at the National Center for Health Statistics (NCHS), and the details can be found elsewhere [9].

The target analytic sample of the present study was adults (≥20 years old) with a history of congestive HF who answered ‘yes’ to the medical question asking if they have ever been diagnosed with congestive HF by a physician or health professional. The multiple cycles of continuous NHANES data were combined to increase the sample size of the target analytic group as well as the statistical precision of the parameter estimates. First, the NHANES data from the four cycles (2007–2008 through 2013–2014) were combined and merged with the mortality data described later. The NHANES 2007–2014 with follow-up mortality data were used to examine the associations of MVPA and SB with all-cause mortality. Two additional cycles were subsequently combined creating the 12-year combined data (2007–2008 through 2017–2018). The NHANES 2007–2018 data were used to examine the prevalence and correlates of MVPA and SB with a larger number of the study sample across the years.

### Exposure variables

During household interviews, NHANES participants completed a PA questionnaire measuring habitual levels of PA and SB. Since the 2007–2008 cycle, the NHANES utilized the Global Physical Activity Questionnaire developed by the World Health Organization [10], which has been extensively applied worldwide with well-established evidence of validity and reliability from different population groups [11]. Moderate- (MPA) and vigorous-intensity physical activity (VPA) were defined as “activities that required moderate physical efforts and cause small increases in breathing or heart rate” and “activities that require hard physical efforts and cause large increases in breathing or heart rate”, respectively. The participants were asked to disclose frequency and duration of PA they have engaged at moderate- or vigorous-intensity levels in a typical week that lasted for at least 10 consecutive minutes across two domains; 1) work (e.g., paid or unpaid work, household chores, yard work); and 2) leisure time-related (e.g., sports, fitness, recreational activities). Additional information about the frequency and duration of transportation-related physical activities such as walking or bicycling they used to travel to and from places in a typical week were obtained, where transportation-related activities were defined as MPA with MET score of 4.0.

Total time spent in MVPA per week was calculated for each participant by adding weekly minutes of MPA and VPA across the three domains (work-, leisure time-related, and transportation), where one minute of VPA was counted as 2 minutes of MPA. Participants were categorized into the three groups, ‘inactive’ for those who did not engage in any MVPA in a typical week (No-MVPA), ‘insufficient’ for those who engaged in some MVPA in a typical week (I-MVPA), but did not meet the current recommendation (ie, ≥150 minutes of MVPA per week), and ‘sufficient’ for those who met the recommended level of MVPA (S-MVPA).

Time spent in SB was obtained from the question asking the participant about the time they have usually spent sitting or in a reclining posture on a typical day for any purposes other than sleeping (e.g., sitting at a desk, traveling in a car, bus, reading, watching television, using a computer). Daily time spent in SB was categorized into SB<8 hours/day vs. SB≥8 hours/day, which is a frequently reported cutoff related to increased risk of adverse clinical health outcomes [8].

### Outcome ascertainment

The follow-up mortality data of the continuous NHANES 2007–2014 were obtained from the 2015 Public-Use Linked Mortality Files provided by the NCHS in CDC. The NCHS utilized probabilistic matching to link the mortality records from the National Death Index with the respective NHANES participants based on various identifying information such as social security number, names, sex, race, etc. [12]. The linked mortality data provide the mortality status of the NHANES participants through 31 December 2015 and follow-up time from the date of household interview until the date of death or the end of the mortality period.

### Study covariates

The following variables were extracted as covariates in consultation with the literature [13, 14]: the time since first diagnosed with congestive HF (<5 and ≥5 years), age (<65 and ≥65 years old), sex (male and female), race/ethnicity (non-Hispanic White, Non-Hispanic black, Mexican American, and others such as other Hispanic, Asian, and multi-racial), education (<high school, high school or equivalent, and ≥College or above), household income (<$25k, $25k-<$75k, and ≥$75k), marital status (married or living with a partner, and others such as widowed, divorced, separated, never married), current smoking status (yes and no), body mass index (BMI<25 kg/m^2^, 25–29.9 kg/m^2^, and ≥30 kg/m^2^), and self-reported chronic medical conditions including coronary heart disease, cancer, hypertension, dyslipidemia, and diabetes. Additionally, the perceived walking difficulty score was calculated by averaging the self-reported responses (4-point Likert scale) to the three mobility limitation questions: 1) walking for a quarter mile difficulty; 2) walking up ten steps difficulty; and 3) walking between rooms on same floor difficulty. The intake of prescribed HF medications (yes and no) was extracted using the self-reported prescription medication data that were coded and classified using the Lexicon Plus® database (Cerner, Multum, Inc.) in the NHANES. Lastly, we calculated the 2015 Heathy Eating Index scores for each participant based on the 24-hour dietary recall interview data, as outlined by elsewhere [15].

### Statistical analysis

Descriptive statistics were estimated using mean or percentage along with the 95% confidence intervals (CI) for continuous and categorical variables, respectively. Using the NHANES 2007–2014 with follow-up mortality data, a Cox proportional hazards regression analysis was conducted. We first fitted the single model examining independent associations of MVPA and SB with all-cause mortality. Study covariates were adjusted in the model using backward elimination, in which the covariates with the largest *P*-value were sequentially removed until the final model consisted of the covariates with *P* < .20. The hazard ratios (HR) and 95% CI were estimated to indicate the relative risk of all-cause death by different levels of MVPA and SB. A linear trend was tested using an orthogonal polynomial contrast for MVPA levels. Joint associations were examined in follow-up analysis where the combined variable with 6 groups (ie, SB with 2 categories x MVPA with 3 categories) was included in the model. Lastly, stratified analyses examining the association of MVPA with all-cause mortality within each category of SB (≤8 hours/day and >8 hours/day) were performed. The multiplicative interaction effect between MVPA and SB on all-cause mortality was tested using a Wald test. The proportional hazard assumption was tested for each variable included in the model using log-log survival curves, Schoenfeld residuals, and by including an interaction term with time in the model. Sensitivity analyses were performed after further excluding the patients who died within two years of follow-up (*n* = 49) to examine the potential bias due to reverse causation.

Using the NHANES 2007–2018 data, cross-sectional analyses were performed to examine the correlates of MVPA and SB based on study covariates including demographic characteristics and previous medical history. Three separate logistic regression models were established predicting the likelihood of engaging in S-MVPA, being inactive (No-MVPA), and engaging in SB≥8 hours/day, respectively. All statistical analyses were conducted using the SAS v9.4 (SAS Institute, Inc., Cary, NC, USA). The SAS PROC SURVEY procedures were used to account for the complex sampling design of the NHANES. The recalculated 8- and 12-year sampling weights were applied for the analyses of the NHANES 2007–2014 mortality data and NHANES 2007–2018 data, respectively.

## Results

### Baseline characteristics

Demographic characteristics of study populations for each cycle are presented in **Online Table 1 in S1 File**. A total of 1,175 adults with HF were identified across the NHANES cycles between 2007 and 2018. Of these, 6 individuals were excluded due to missing and/or invalid responses to study variables and the remaining sample of 1169 was used for cross-sectional analysis examining the population-level prevalence and correlates of MVPA and SB. The data from the first four cycles (2007–2008 through 2013–2014) were eligible to link with follow-up mortality data. Of 756 mortality eligible samples, 45 were excluded due to early death (<1 year) after follow-up to reduce the risk of bias from reverse causation, resulting in the final analytic sample of 711 for survival analysis for all-cause mortality. The flow diagram of the selection of the analytic sample is presented in **Online Fig 1 in S1 File**.

Descriptive characteristics of the study population estimated from the NHANES 2007–2014 with follow-up mortality data are presented in **Table 1**. Additional descriptive tables stratified by MVPA and SB levels are reported in **Online Tables 2** - **3 in S1 File**, respectively. Approximately 2.32% (95% CI = 2.05, 2.59) of the US noninstitutionalized adult population had a history of HF. The majority were older adults aged ≥65 years (61.54%; 95% CI = 56.21, 66.86) and had been diagnosed with HF ≥5 years ago (63.62%; 95% CI = 57.79, 69.46).

**Table 1:** Descriptive characteristics of the US adults with a history of congestive heart failure (NHANES 2007–2014).

	NHANES 2007–2014
Unweighted n	711
Weighted n	5,085,409
Weighted %^a^	2.32 (2.05, 2.59)
Years since the first diagnose with HF	
Mean (years)	8.96 (8.09, 9.83)
<5 years	36.38 (30.54, 42.21)
≥5 years	63.62 (57.79, 69.46)
Age	
Mean (years)	65.54 (64.24, 66.83)
<65 years	38.47 (33.14, 43.79)
≥65 years	61.54 (56.21, 66.86)
Sex	
Male	49.12 (44.19, 54.05)
Female	50.88 (45.95, 55.81)
Race/ethnicity	
Non–Hispanic white	70.89 (65.94, 75.84)
Non–Hispanic black	15.39 (11.94, 18.84)
Mexican American	4.62 (2.25, 6.99)
Others	9.10 (6.10, 12.11)
Education level	
<High school	30.91 (26.24, 35.59)
High school	26.59 (22.79, 30.38)
≥College or above	42.50 (37.72, 47.28)
Household income	
<$25k	40.06 (34.97, 45.14)
$25k - <$75k	47.40 (41.65, 53.15)
≥$75k	12.54 (8.70, 16.39)
Marital status	
Married or partner	57.29 (52.00, 62.59)
Smoking status	
Currently smoking	19.69 (15.33, 24.06)
Body mass index	
<25 kg/m^2^	24.41 (20.32, 28.49)
25–29.9 kg/m^2^	24.54 (20.70, 28.38)
≥30 kg/m^2^	51.06 (45.66, 56.45)
Chronic medical conditions (yes)	
Coronary heart disease	35.56 (31.06, 40.07)
Cancer	24.44 (19.58, 29.29)
High blood pressure	77.57 (73.62, 81.52)
High cholesterol	63.30 (58.44, 68.17)
Diabetes	38.93 (34.13, 43.73)
Walking difficulty score (Mean; 95% CI) ^b^	1.49 (1.43, 1.57)
HF medications (yes)	83.49 (79.68, 87.31)
Beta blockers	62.38 (57.56, 67.21)
ACEI or ARBNI	36.55 (31.63, 41.46)
Aldosterone antagonist	7.55 (5.07, 10.02)
Vasodilators	8.89 (6.46, 11.32)
Diuretics	48.84 (43.47, 54.21)

HF = heart failure; CI = confidence interval; ACEI = angiotensin converting enzyme inhibitor; ARBNI = angiotensin II receptor blocker neprilysin inhibitor

Values the percentage (95% CI) unless otherwise specified.

^a^ the weighted proportion of the individuals with HF among the US adults

^b^ walking difficulty score is an average score of the three self-reported questions with 4-points Likert type scale.

### Independent effects of MVPA and SB on all-cause mortality

**Table 2** presents the results of Cox proportional hazard regression analyses examining the independent associations of MVPA and SB with all-cause mortality. Both MVPA and SB were independently associated with all-cause mortality after adjusting for study covariates. A significant linear trend for MVPA levels was observed (*P*-for-trend < .001), in which HF patients who were in S-MVPA (HR = 0.53; 95% CI = 0.31, 0.89) had lower risk of mortality when compared to No-MVPA group. There was no significance difference in the risk of morality between I-MVPA and S-MVPA (HR = 0.79; 95% CI = 0.41, 1.54). Patients with HF and SB≥8 hours/day had greater risk of mortality (HR = 1.59; 95% CI = 1.07, 2.36) when compared to those with SB<8 hours/day.

**Table 2:** Independent associations of MVPA and sedentary time with all-cause mortality among the US adults with a history of congestive heart failure (NHANES 2007–2014).

	Weighted %	Hazard ratio (95% CI)^a^	
	(95% CI)		
Unweighted *n* (deaths)	711 (199)	-	
Weighted *n* (deaths)	5,085,409	-	
	(1,245,543)		
MVPA (3 categories)			
No-MVPA	51.48%	referent	-
	(46.70, 56.27)		
I-MVPA	15.24%	0.67	referent
	(12.36, 18.11)	(0.41, 1.09)	
S-MVPA	33.28%	0.53*	0.79
	(28.59, 37.98)	(0.31, 0.89)	(0.41, 1.54)
P-for-trend		< .001	
Sedentary time			
<8 hours/day	50.56%	referent	
	(46.29, 54.84)		
≥8 hours/day	49.44%	1.59*	
	(45.16, 53.71)	(1.07, 2.36)	

^a^ the estimates were obtained from the Cox proportional hazard regression model adjusting for the study covariates retained using the backward elimination approach (*P* < .20). The covariates retained in both models included age group, race/ethnicity, education, BMI group, marital status, and self-reported medical conditions on coronary heart disease, high cholesterol, diabetes, and walking difficulty score.

### Joint effects of MVPA and SB on all-cause mortality

**Fig 1:**
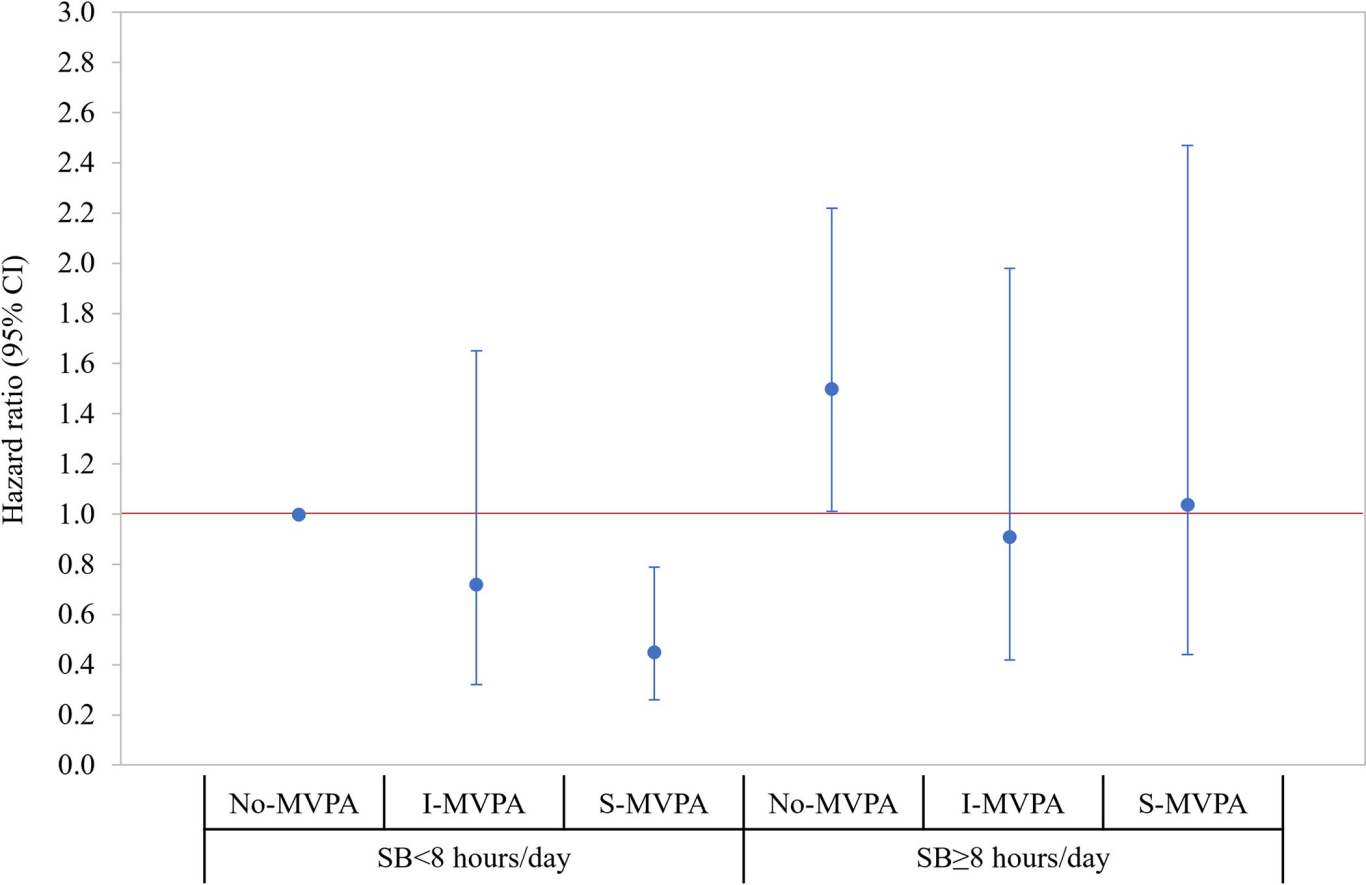
Joint association where HF patients with SB<8 hours/day associated with inactive (no-MVPA) were a reference group. Hazard ratios (95% CI) were estimated from the Cox proportional hazard regression model adjusting the study covariates retained using the backward elimination approach (*P* < .20), which include age group, race/ethnicity, education, BMI group, marital status, and self-reported medical conditions (coronary heart disease, high cholesterol, and diabetes), and walking difficulty score. The estimated parameters presented in the figures are reported in **Online Supplemental Table 4 in S1 File**. CI = confidence interval; SB = sedentary behavior; I-MVPA = insufficient moderate-vigorous-intensity physical activity (MVPA minute/week between 0 and 150); S-MVPA = sufficient moderate- and vigorous-intensity physical activity (≥150 minutes/week of MVPA).

**Fig 2:**
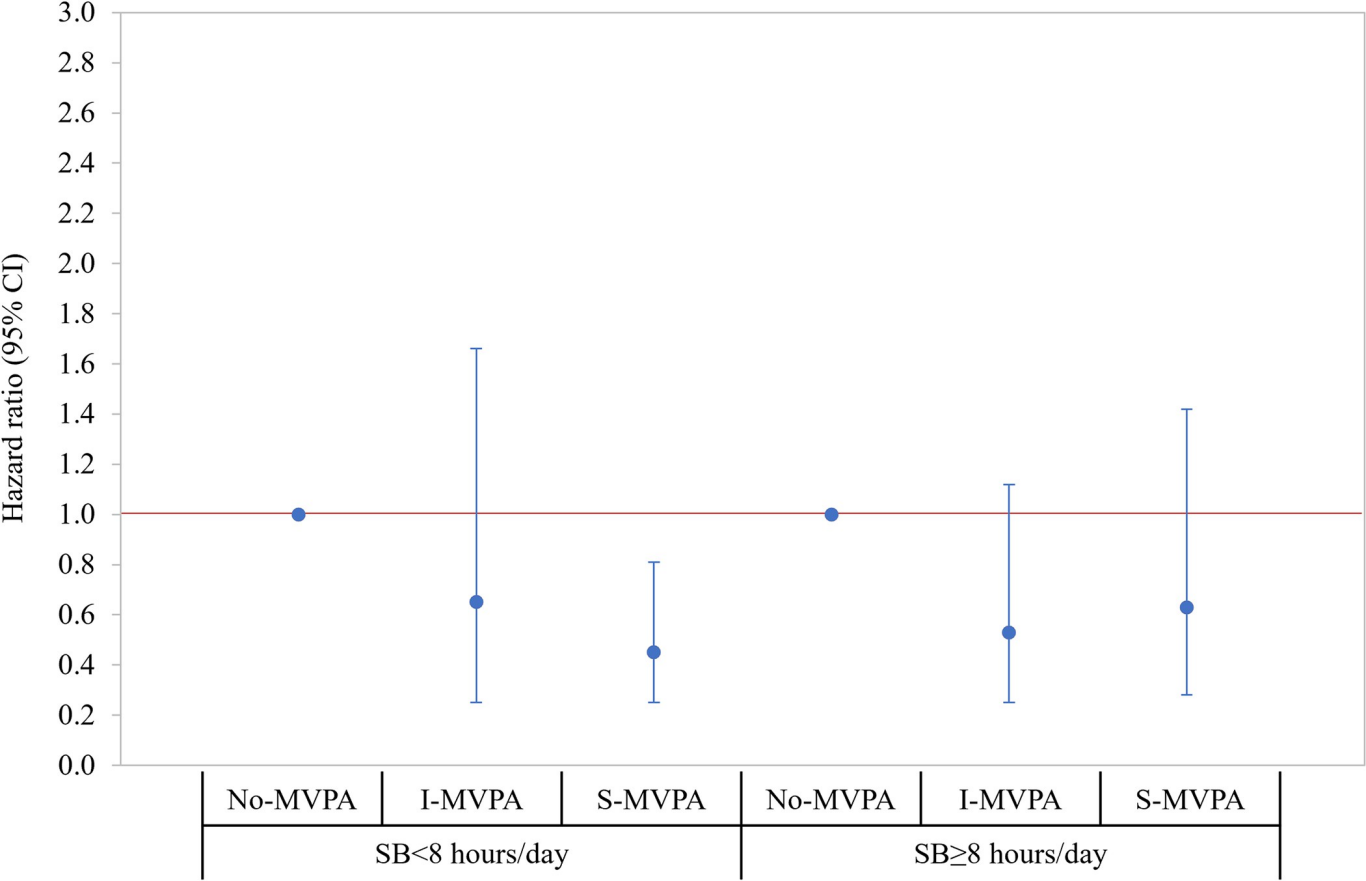
Stratified associations by SB level where HF patients with inactive (no-MVPA) were a reference group within each stratum. Hazard ratios (95% CI) were estimated from the Cox proportional hazard regression model adjusting the study covariates retained using the backward elimination approach (*P* < .20), as described in **Fig 1**. The estimated parameters presented in the figures are reported in **Online Supplemental Table 5 in S1 File**. CI = confidence interval; SB = sedentary behavior; I-MVPA = insufficient moderate-vigorous-intensity physical activity (MVPA minute/week between 0 and 150); S-MVPA = sufficient moderate- and vigorous-intensity physical activity (≥150 minutes/week of MVPA).

**Fig 3:**
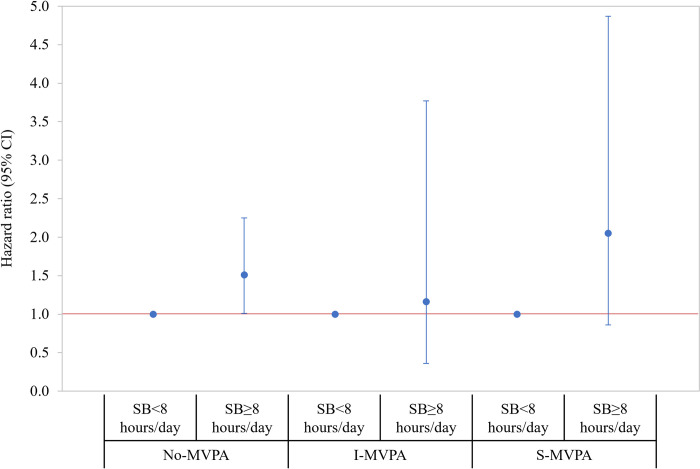
Stratified associations by MVPA level where HF patients with SB<8 hours/day were a reference group within each stratum. Hazard ratios (95% CI) were estimated from the Cox proportional hazard regression model adjusting the study covariates retained using the backward elimination approach (*P* < .20), as described in **Fig 1**. The estimated parameters presented in the figures are reported in **Online Supplemental Table 6 in S1 File**. CI = confidence interval; SB = sedentary behavior; I-MVPA = insufficient moderate-vigorous-intensity physical activity (MVPA minute/week between 0 and 150); S-MVPA = sufficient moderate- and vigorous-intensity physical activity (≥150 minutes/week of MVPA).

**Fig 1** presents the joint association of MVPA and SB with all-cause mortality. Using HF patients with SB<8 hours/day associated with No-MVPA as a reference group, there was a significantly lower risk of mortality among those with SB<8 hours/day associated with S-MVPA (HR = 0.45; 95% CI = 0.26, 0.79) and greater risk of mortality among those with SB≥8hours/day associated with No-MVPA (HR = 1.50; 95% CI = 1.01, 2.22). Additionally, the latter group (SB≥8 hours/day associated with No-MVPA) had a greater risk of mortality when compared to those in all other groups (**Online Table 4 in S1 File**). The results of stratified analyses are presented in **Figs 2 and 3**, respectively. When stratified by SB level, the risk of mortality was decreased by increasing the level of MVPA (*P*-for-trend = .009) among HF patients with SB<8hours/day. Of note, MVPA was not significantly associated with the risk of mortality among those with SB≥8 hours/day (**Online Table 5 in S1 File)**. When stratified by MVPA level, patients with SB≥8 hours/day had a greater risk of mortality (HR = 1.51; 95% CI = 1.01, 2.25) when compared to those with SB<8 hours/day in No-MVPA group only and there was no statistically significant difference in the relative risk of mortality by SB levels among those in the I- and S-MVPA groups (**Online Table 6 in S1 File**). The interaction effect between MVPA and SB was not statistically significant (Wald *x*^2^ = 0.68; *df* = 2; *P* = .711).

### Prevalence and correlates of MVPA and SB in HF patients

The prevalence of MVPA and SB based on the 12-year combined NHANES data (2007–2014) are presented in **Online Tables 7–9 in S1 File**. The results of multivariate logistic regression analyses examining the demographic correlates of MVPA and SB are presented in **Table 3**. Model 1 demonstrated that being male (OR = 0.69; 95% CI = 0.47, 0.99), having lower education level (OR = 0.63; 95% CI = 0.42, 0.96) and having cancer (OR = 0.59; 95% CI = 0.37, 0.94), were significant predictors associated with lower odds of meeting S-MVPA levels. Additional analysis predicting the likelihood of being No-MVPA (Model 2) showed that having lower education and household income levels (*P*-for-trends = .02 and .01, respectively) and having diabetes (OR = 1.43; 95% CI = 1.02, 2.02) were significantly associated with the greater odds of being No-MVPA.

**Table 3:** The correlates of MVPA and sedentary behavior among adults with a history of congestive heart failure (NHANES 2007–2018).

	Multivariate logistic regression analysis		
	Model 1: S-MVPA ^a^	Model 2: No-MVPA ^b^	Model 3: SB≥8 hours/day ^c^
	(reference: No-MVPA + I-MVPA)	(reference: I-MVPA + S-MVPA)	(reference: <8 hours/day)
Years since the first diagnose with HF			
<5 years	0.70 (0.46, 1.06)	1.48 (0.99, 2.21)	1.59* (1.15, 2.19)
≥5 years	(reference)	(reference)	(reference)
Age			
<65 years	0.92 (0.60, 1.42)	1.17 (0.75, 1.81)	1.37 (0.92, 2.04)
≥65 years	(reference)	(reference)	(reference)
Sex			
Male	0.69* (0.47, 0.99)	1.39 (0.96, 2.01)	1.11 (0.80, 1.55)
Female	(reference)	(reference)	(reference)
Race/ethnicity			
Non-Hispanic white	(reference)	(reference)	(reference)
Non-Hispanic black	0.69* (0.49, 0.98)	1.22 (0.84, 1.77)	0.91 (0.63, 1.32)
Mexican American	1.23 (0.70, 2.13)	0.90 (0.55, 1.45)	0.78 (0.38, 1.59)
Others	0.92 (0.56, 1.50)	0.99 (0.65, 1.53)	0.78 (0.46, 1.32)
Education level			
<High school	0.63* (0.42, 0.96)	1.59* (1.10, 2.31)	0.93 (0.60, 1.44)
High school	1.04 (0.64, 1.69)	1.10 (0.70, 1.72)	1.20 (0.77, 1.87)
≥College or above	(reference)	(reference)	(reference)
*P*-for-trend	.09	.02*	.73
Household income			
<$25k	0.61 (0.32, 1.17)	1.92* (1.15, 3.20)	0.55* (0.33, 0.91)
$25k –<$75k	0.62 (0.32, 1.22)	1.92* (1.13, 3.28)	0.63 (0.37, 1.06)
≥$75k	(reference)	(reference)	(reference)
*P*-for-trend	.14	.01*	.02*
Marital status			
Married or partner	(reference)	(reference)	(reference)
Others	0.90 (0.58, 1.39)	0.98 (0.66, 1.46)	1.25 (0.90, 1.75)
Smoking status			
Currently smoking	1.18 (0.78, 1.77)	0.77 (0.50, 1.20)	1.01 (0.61, 1.67)
Not smoking	(reference)	(reference)	(reference)
Body mass index			
<25 kg/m^2^	(reference)	(reference)	(reference)
25–29.9 kg/m^2^	1.10 (0.61, 1.97)	0.72 (0.45, 1.18)	1.14 (0.72, 1.81)
≥30 kg/m^2^	1.06 (0.64, 1.76)	0.90 (0.58, 1.39)	1.80* (1.13, 2.86)
*P*-for-trend	.82	.62	.01*
Chronic medical conditions (yes)			
Coronary heart disease	0.99 (0.68, 1.48)	1.19 (0.83, 1.73)	1.43* (1.06, 1.93)
Cancer	0.59* (0.38, 0.94)	1.22 (0.75, 1.96)	0.80 (0.53, 1.22)
High blood pressure	0.75 (0.48, 1.17)	0.94 (0.62, 1.42)	0.82 (0.50, 1.34)
High cholesterol	1.29 (0.88, 1.88)	0.89 (0.63, 1.26)	0.86 (0.58, 1.28)
Diabetes	0.74 (0.48, 1.13)	1.43* (1.02, 2.02)	1.17 (0.81, 1.69)
Walking difficulty score (Mean; 95% CI)	0.54* (0.41, 0.71)	2.04* (1.55, 2.68)	1.30* (1.05, 1.60)
HF medications (yes)	0.91 (0.51, 1.60)	1.35 (0.84, 2.17)	1.18 (0.75, 1.87)

NHANES = National Health and Nutrition Examination Survey; HF = heart failure

Th complex sampling design of the NHANES was applied 12-year sampling weights. Values are odds ratios (95% confidence intervals) estimated from the logistic regression model including all study variables.

^a^ model 1 estimated the likelihood of being ‘S-MVPA’ using (No-MVPA + I-MVPA) group as a reference.

^b^ model 2 estimated the likelihood of being ‘No-MVPA’ using (I-MVPA + S-MVPA) group as a reference.

^c^ model 3 estimated the likelihood of being ‘≥8 hours/day of sedentary time’ using ‘<8 hours/day’ group as a reference.

* *P* < .05

Lastly, Model 3 predicting the likelihood of being SB≥8 hours/day demonstrated that having <5 years since the first diagnosis of HF (OR = 1.59; 95% CI = 1.15, 2.19), greater BMI levels (*P*-for-trend = .01), and having coronary heart disease (OR = 1.43; 95% CI = 1.06, 1.93) were significantly associated with greater odds of SB≥8 hours/day; moreover, lower household income was significantly associated with lower odds of SB≥8 hours/day (*P*-for-trend = .02).

## Discussion

To the best of our knowledge, this study is one of the few studies examining the associations of MVPA and SB with all-cause mortality in patients with established HF. The results showed that greater MVPA and lower levels of SB are both independently associated with reduced risk of all-cause mortality in HF patients. Among MVPA categories, both I-MVPA and S-MVPA were associated with a significantly lower risk (ie, reductions of 37% and 47% for all-cause mortality, respectively) compared to No-MVPA group (ie, not engaging in any MVPA). This suggests that in HF patients, MVPA, even when lower than what is recommended by the current PA guidelines (ie, ≥150 minutes of MVPA/week), still provides substantial benefits compared to those who are physically inactive. This resonates with the overarching message of the current US PA guidelines–move more and sit less [4]. Independent of MVPA levels, when HF patients spent ≥8 hours/day in SB, the relative risk for all-cause death increased by 67%, highlighting the clinical independent importance of reducing SB throughout the day in patients with HF.

As expected, when taking both MVPA and SB into consideration, HF patients with S-MVPA and SB<8 hours/day demonstrated the lowest risk of all-cause mortality; yet, few notable variations were observed by different combinations of MVPA and SB levels. First, the mortality risk among patients with I-MVPA and SB<8 hours/day was not significantly different compared to patients with S-MVPA and SB<8 hours/day. Furthermore, the beneficial effect of MVPA was apparent with a significant linear trend (*P* < .001) among patients with SB<8 hours/day; yet, this effect was less pronounced among patients with SB≥8 hours/day. Although we cannot entirely rule out the possibility of the results being underpowered given relatively small sample size and the size of effects, the present findings generally suggest that even a lower engagement in PA, below the current ideal PA recommendations, would provide beneficial effects on survival in HF patients when combined with SB<8 hours/day. However, with SB≥8 hours/day, the beneficial effects of MVPA on mortality may be attenuated, even after meeting current ideal PA recommendations.

On the other hand, being No-MVPA and having SB≥8 hours/day was associated with a profound increase in mortality risk, which was 161% higher when compared to patients with No-MVPA but SB<8 hours/day. This finding indicates that reducing SB to <8 hours/day in HF patients would have beneficial effects, even in those who do not routinely engage in MVPA. The stratified analyses by MVPA levels provided additional insights in that the risk of mortality associated with SB was not statistically significant among patients with I- or S-MVPA levels. This supports the notion that in patients with established HF, engaging in some level of MVPA may play a critical role in attenuating detrimental effects of SB on longevity.

Our results are aligned with an existing body of evidence supporting MVPA as an independent risk factor associated with mortality among HF patients. For instance, using the prospective cohort data among 902 HF patients, Doukky et al. [16] showed that the risk of all-cause death increased with decreasing PA levels. Particularly, they highlighted that even modest exercise (≥1 minute per week) provides survival benefit in this clinical population, which are consistent with the present findings. Meanwhile, there is still a paucity of literature concerning the impact of SB on longevity among HF patients. Of the limited empirical data, Doukky, et al. [16] also reported greater time spent in SB, represented by self-reported television viewing time, was independently associated with an increased risk of all-cause death. Another study based on a multi-site cohort study of 602 HF patients concluded a self-reported sedentary lifestyle was associated with an increased risk of all-cause mortality [17].

The clinical benefits of PA on CV health and physiological pathways linking to improved longevity in HF patients are well documented [18]. However, the biological response to SB is only vaguely understood, which is known to be complex, involving multiple factors such as, peripheral organs regulating energy metabolism (e.g., skeletal muscle, adipose tissue), the sympathetic nervous system, and oxidative stress and inflammatory pathways. However, the mechanistic evidence linking SB to CV health remains scarce [19]. This indicates that in HF patients, the pathophysiological effects of SB could be more complex due to several HF-related impairments or alterations in energy metabolism or nervous system function [20, 21]. Nevertheless, the finding of the present study supports the growing evidence demonstrating the harmful independent effects of excessive SB on longevity in this clinical population.

In addition, there has been a growing interest about the interactive role of MVPA and SB [8, 22], and recent PA guidelines highlight the potential beneficial effects of MVPA on reducing or even eliminating the health risk associated with high SB [23]. For instance, the World Health Organization recommends adults living with chronic conditions (ie, hypertension, type 2 diabetes, HIV, and cancer survivors) engaging in more than the recommended levels of MVPA (≥150 minutes per week) to also avoid high SB to further reduce the detrimental effects associated with this unfavorable lifestyle characteristic [24]. However, the evidence was based on studies from the general population and there is little data on the effects of SB from clinical populations, such as HF. In this regard, the present study adds important results to the body of literature in that, among patients with established HF, engaging in any amount of MVPA would provide significant health benefits by reducing the risk of mortality associated with high SB (ie, ≥8 hours/day). Furthermore, our study demonstrated that engaging in SB≥8 hours/day would attenuate potential beneficial effects associated with MVPA, which collectively supports the notion that greater health benefits would be expected by both engaging in MVPA and reducing SB among HF patients.

In line with previous studies [25], the present study demonstrated that HF patients had lower levels of MVPA and higher levels of SB when compared to those without HF (**Online Fig 2 in S1 File**). We expanded our findings to investigate the correlates for MVPA and SB that can be targeted in a future intervention study. The results highlight that MVPA and SB in HF patients are largely influenced by socioeconomic status and types of chronic diseases. We found that men, lower education level, and the presence of cancer were the strongest predictors for not meeting S-MVPA, while lower education level, lower household income, and the presence of diabetes were associated with the higher likelihood of being No-MVPA. Finally, having had a diagnosis of HF within 5 years, in addition to lower household income, having a higher BMI, and coronary heart disease were associated with a higher likelihood of participating in SB≥8 hours/day. The present findings provide additional insight into the potential demographic correlates leading to health disparities in HF patients. However, given that PA and SB are complex lifestyle behaviors determined by multiple individual- and contextual-level factors, future research should focus on identifying HF-specific modifiable factors and the development of intervention strategies to promote healthy lifestyle behaviors in HF patients.

Our study is not without limitations. First, the diagnosis of HF was self-reported without the distinction of HF phenotypes. Certainly, the data demonstrating the protective effects of PA to prevent HF with preserved ejection fraction is stronger than for HF with reduced ejection fraction, although substantial data supports the benefits of PA in both types of established HF [26]. Additionally, clinically relevant information, such as severity of symptoms, functional classification, hospitalizations, types of treatment received, etc., was not available. Such limited covariate may further introduce the residual and unmeasured confounding. Secondly, both MVPA and SB were not objectively measured but rather reported by the patients at baseline. This implies that the results might be affected by the potential self-reported measurement errors (e.g., recall bias), which may, in part, be responsible for a statistically non-significant multiplicative interaction effect between SB and MVPA. Future research is encouraged to examine the time-dependent and dose-response effects of objectively measured MVPA and context-specific SB. Also, the follow-up duration of the NHANES is relatively short which may introduce the bias due to reverse causation. Although the present study excluded early deaths (<1 year after follow-up) and the results of sensitivity analyses after further excluding the deaths within two years of follow-up did not significantly alter the primary results, we cannot fully rule out the risk of bias due to reverse causation. Finally, our study could not investigate the effects of SB and MVPA on top of current guideline-directed medical therapy since in the last 5 years there have been significant improvements in the treatment of patients with HF, and our population was enrolled prior to the implementation of these changes. Specifically, we believe that future studies should possibly replicate our analysis in patients who are on current guideline-directed medical therapy, including newer agents with proven benefits in this population, such as angiotensin receptor-neprilysin inhibitors and sodium-glucose co-transporter 2 inhibitors. Of note, the most recent HF Guidelines [27] do not provide specific recommendation with regards to SB nor MVPA in patients with established HF, therefore our study provides novel information that will hopefully inform future guidelines and recommendations from scientific societies with the ultimate goal of improve patients’ outcomes.

In conclusion, using a nationally representative sample of US adults, the present study suggests that any level of MVPA could provide modest benefits on survival in patients with established HF; however, these benefits can be attenuated by excessive SB (ie, ≥8 hours/day). Moreover, in those who had No-MVPA, lower SB (ie, <8 hours/day) was associated with reduced mortality, suggesting SB could be an independent therapeutic target when increasing MVPA is not a feasible option due to the symptom-related barriers (e.g., fatigue, pain) or other factors. Finally, we have identified several correlates of PA, which, when modifiable, could be targeted to promote a greater level of MVPA and lower SB. Further prospective randomized trials aimed at increasing PA and reducing SB in patients with established HF, particularly in the groups we have identified as higher risk for not meeting MVPA and SB levels required to improve survival, are urgently needed to confirm and expand our findings.

## Supporting information

S1 File Online supplemental materials. (DOCX)

## Abbreviation and acronyms

BMI: body mass index
CDC: Centers for Disease Control and Prevention
CI: confidence interval
CV: cardiovascular
CVD: cardiovascular disease
HF: heart failure
HR: hazard ratio
I-MVPA: insufficient MVPA (<150 minutes of MVPA per week)
MPA: moderate-intensity physical activity
MVPA: moderate- and vigorous-intensity physical activity
NCHS: National Center for Health Statistics
NHANES: National Health Nutrition Examination Survey
No-MVPA: no engagement in MVPA
PA: physical activity
SB: sedentary behavior
S-MVPA: sufficient MVPA (≥150 minutes of MVPA per week)
US: United States
VPA: vigorous-intensity physical activity
