# Supplementary material for: Physical activity, sedentary behaviors and all-cause mortality in patients with heart failure: Findings from the NHANES 2007–2014
Source: PLoS One. 2022 Jul 15;17(7):e0271238. doi: 10.1371/journal.pone.0271238 (PMC9286289; doi:10.1371/journal.pone.0271238)

**Online Table 1.**

|  | Continuous NHANES Cycles | | | | | |
| --- | --- | --- | --- | --- | --- | --- |
|  | 2007-2008 | 2009-2010 | 2011-2012 | 2013-2014 | 2015-2016 | 2017-2018 |
| Unweighted n | 214 | 173 | 187 | 182 | 213 | 200 |
| Weighted n | 5,058,161 | 4,325,669 | 6,112,484 | 5,960,260 | 5,784,267 | 5,461,903 |
| Weighted % (95% CI) | 2.36  (1.79, 2.94) | 1.98  (1.50, 2.47) | 2.74  (2.01, 3.47) | 2.61  (2.26, 2.96) | 2.48  (2.04, 2.91) | 2.30  (1.66, 2.93) |
| Years since the first diagnose with HF | | | | | | |
| <5 years | 31.83  (22.81, 40.84) | 49.11  (34.49, 63.73) | 41.31  (30.08, 52.55) | 30.56  (19.23, 41.89) | 39.77  (30.38, 49.17) | 34.97  (25.87, 44.06) |
| ≥5 years | 68.17  (59.16, 77.19) | 50.89  (36.27, 65.52) | 58.69  (47.45, 69.92) | 69.44  (58.11, 80.77) | 60.23  (50.83, 69.62) | 65.03  (55.94, 74.13) |
| Age |  |  |  |  |  |  |
| <65 years old | 36.75  (30.69, 42.82) | 43.72  (35.23, 52.21) | 32.59  (19.76, 45.42) | 39.75  (31.25, 48.25) | 32.09  (23.34, 40.84) | 43.26  (31.97, 54.55) |
| ≥65 years old | 63.25  (57.18, 69.31) | 56.28  (47.79, 64.77) | 67.41  (54.58, 80.24) | 60.25  (51.75, 68.75) | 67.91  (59.16, 76.66) | 56.74  (45.45, 68.03) |
| Sex |  |  |  |  |  |  |
| Male | 53.56  (43.88, 63.23) | 54.73  (47.80, 61.67) | 42.89  (33.45, 52.32) | 46.57  (36.23, 56.9) | 52.82  (46.06, 59.59) | 59.93  (52.87, 66.99) |
| Female | 46.44  (36.77, 56.12) | 45.27  (38.33, 52.2) | 57.11  (47.68, 66.55) | 53.43  (43.1, 63.77) | 47.18  (40.41, 53.94) | 40.07  (33.01, 47.13) |
| Race/ethnicity |  |  |  |  |  |  |
| Non-Hispanic white | 65.14  (51.83, 78.45) | 73.71  (62.19, 85.23) | 71.47  (64.52, 78.41) | 73.50  (66.17, 80.83) | 60.89  (51.34, 70.43) | 69.39  (59.33, 79.44) |
| Non-Hispanic black | 18.01  (10.61, 25.41) | 16.86  (8.49, 25.24) | 12.95  (6.48, 19.43) | 14.81  (10.4, 19.23) | 17.95  (10.85, 25.05) | 15.34  (7.42, 23.26) |
| Mexican American | 3.37  (1.27, 5.48) | 3.16  (0.40, 5.92) | 4.24  (0, 9.42) | 6.58  (1.41, 11.75) | 5.56  (2.10, 9.02) | 3.61  (0.94, 6.27) |
| Others | 13.48  (7.10, 19.85) | 6.26  (2.50, 10.03) | 11.34  (2.78, 19.90) | 5.11  (2.20, 8.03) | 15.60  (10.86, 20.34) | 11.67  (5.85, 17.49) |

**Descriptive Characteristics of the US Adults with a History of Congestive Heart Failure (NHANES 2007 – 2018)**

**Online Table 1 (continue).**

**Descriptive Characteristics of the US Adults with a History of Congestive Heart Failure (NHANES 2007 – 2018)**

|  | Continuous NHANES Cycles | | | | | |
| --- | --- | --- | --- | --- | --- | --- |
|  | 2007-2008 | 2009-2010 | 2011-2012 | 2013-2014 | 2015-2016 | 2017-2018 |
| Education level |  |  |  |  |  |  |
| <High school | 35.58  (27.57, 43.59) | 30.59  (22.61, 38.57) | 35.12  (23.71, 46.54) | 23.20  (18.04, 28.37) | 23.55  (15.29, 31.81) | 19.23  (12.05, 26.41) |
| High school | 29.71  (24.30, 35.12) | 28.86  (21.73, 35.98) | 23.92  (16.2, 31.63) | 27.81  (20.16, 35.47) | 24.97  (19.23, 30.72) | 35.59  (27.80, 43.39) |
| ≥College or above | 34.71  (23.59, 45.83) | 40.55  (32.41, 48.7) | 40.96  (32.81, 49.11) | 48.99  (41.01, 56.96) | 51.48  (44.71, 58.25) | 45.18  (36.32, 54.03) |
| Household income |  |  |  |  |  |  |
| <$25k | 44.70  (35.75, 53.65) | 32.38  (23.3, 41.46) | 43.08  (31.78, 54.38) | 41.53  (32.55, 50.5) | 47.30  (40.72, 53.88) | 32.80  (26.75, 38.84) |
| $25k – <$75k | 47.26  (37.67, 56.85) | 48.92  (39.03, 58.81) | 46.32  (33.83, 58.81) | 45.21  (33.90, 56.52) | 39.65  (28.39, 50.91) | 45.76  (33.92, 57.59) |
| ≥$75k | 8.04  (1.91, 14.18) | 18.69  (8.57, 28.82) | 10.60  (4.15, 17.05) | 13.26  (6.76, 19.77) | 13.05  (5.17, 20.93) | 21.45  (10.50, 32.4) |
| Marital status |  |  |  |  |  |  |
| Married or partner | 49.61  (35.25, 63.96) | 61.49  (54.24, 68.74) | 55.18  (45.65, 64.71) | 57.11  (50.81, 63.4) | 47.8  (37.58, 58.01) | 52.08  (40.67, 63.49) |
| Others | 50.39  (36.04, 64.75) | 38.51  (31.26, 45.76) | 44.82  (35.29, 54.35) | 42.89  (36.6, 49.19) | 52.20  (41.99, 62.42) | 47.92  (36.51, 59.33) |
| Smoking status |  |  |  |  |  |  |
| Currently smoking | 19.50  (13.93, 25.06) | 17.92  (10.43, 25.4) | 21.70  (9.53, 33.87) | 20.53  (15.26, 25.8) | 17.72  (10.26, 25.19) | 15.06  (9.63, 20.48) |
| Body mass index | | | | | | |
| <25 kg/m^2^ | 29.50  (22.41, 36.59) | 20.95  (15.34, 26.55) | 26.00  (15.01, 36.98) | 27.76  (21.45, 34.07) | 25.51  (17.85, 33.16) | 19.50  (12.76, 26.24) |
| 25 – 29.9 kg/m^2^ | 30.95  (22.27, 39.62) | 21.71  (16.37, 27.04) | 21.59  (17.17, 26) | 21.73  (11.85, 31.6) | 23.17  (17.32, 29.02) | 16.62  (11.37, 21.88) |
| ≥30 kg/m^2^ | 39.55  (33.29, 45.81) | 57.35  (50.07, 64.62) | 52.42  (38.89, 65.95) | 50.51  (41.20, 59.82) | 51.32  (42.81, 59.83) | 63.88  (57.42, 70.34) |

**Online Table 1 (continue).**

**Descriptive Characteristics of the US Adults with a History of Congestive Heart Failure (NHANES 2007 – 2018)**

|  | Continuous NHANES Cycles | | | | | |
| --- | --- | --- | --- | --- | --- | --- |
|  | 2007-2008 | 2009-2010 | 2011-2012 | 2013-2014 | 2015-2016 | 2017-2018 |
| Chronic medical conditions (yes) | | | | | | |
| Coronary heart disease | 37.29  (31.16, 43.42) | 37.30  (27.82, 46.78) | 31.86  (25.88, 37.85) | 37.42  (27.05, 47.80) | 47.30  (36.72, 57.88) | 43.57  (32.63, 54.52) |
| Cancer | 23.87  (12.14, 35.61) | 25.66  (19.62, 31.70) | 18.95  (11.4, 26.49) | 27.8  (17.39, 38.21) | 23.83  (17.31, 30.35) | 23.95  (15.86, 32.03) |
| High blood pressure | 75.5  (68.75, 82.24) | 75.41  (65.49, 85.32) | 81.44  (75.4, 87.47) | 78.48  (70.25, 86.71) | 84.02  (76.15, 91.88) | 79.88  (70.97, 88.79) |
| High cholesterol | 54.92  (47.45, 62.38) | 63.77  (58.73, 68.81) | 64.18  (54.40, 73.97) | 66.99  (55.26, 78.72) | 67.29  (58.27, 76.31) | 51.36  (41.85, 60.88) |
| Diabetes | 41.79  (33.57, 50.01) | 37.96  (28.05, 47.88) | 39.00  (29.52, 48.48) | 38.19  (29.91, 46.47) | 39.91  (30.71, 49.11) | 49.42  (41.13, 57.72) |
| Walking difficulty score (Mean; 95% CI) | 1.57 (1.43, 1.70) | 1.39 (1.21, 1.56) | 1.32 (1.18, 1.46) | 1.41 (1.25, 1.57) | 1.55 (1.41, 1.69) | 1.51 (1.35, 1.67) |
| HF medications (yes) | 79.60  (70.09, 89.11) | 84.68  (78.25, 91.12) | 88.72  (84.41, 93.03) | 82.73  (75.28, 90.18) | 82.80  (76.05, 89.55) | 87.13  (83.51, 90.76) |
| Beta blockers | 57.15  (46.23, 68.07) | 67.58  (58.52, 76.64) | 62.59  (55.64, 69.54) | 66.40  (57.48, 75.32) | 67.32  (58.71, 75.94) | 71.05  (64.58, 77.53) |
| ACEI or ARBNI | 29.87  (21.63, 38.10) | 40.00  (31.13, 48.87) | 40.37  (29.06, 51.67) | 34.85  (28.73, 40.97) | 29.68  (19.38, 39.98) | 38.93  (29.20, 48.66) |
| Aldosterone antagonist | 6.30  (3.26, 9.33) | 9.55  (2.45, 16.65) | 4.71  (1.13, 8.28) | 10.83  (6.45, 15.21) | 5.85  (0.76, 10.95) | 12.95  (6.30, 19.60) |
| Vasodilators | 6.03  (2.79, 9.28) | 6.73  (3.27, 10.20) | 14.20  (7.01, 21.39) | 7.89  (4.58, 11.20) | 9.55  (4.47, 14.64) | 9.79  (4.40, 15.18) |
| Diuretics | 47.11  (39.82, 54.39) | 51.15  (41.98, 60.33) | 49.00  (36.30, 61.71) | 52.85  (43.78, 61.93) | 44.81  (38.29, 51.33) | 53.32  (42.59, 64.04) |

HF = heart failure; NHANES = National Health and Nutrition Examination Survey; ACEI = angiotensin converting enzyme inhibitor; ARBNI = angiotensin II receptor blocker neprilysin inhibitor

Values are the percentages (95% CI) unless otherwise specified. A 2-years sampling weight was applied for each cycle.

**Online Table 2.**

**Descriptive Characteristics of the Study Populations by MVPA Groups (NHANES 2007 - 2014)**

|  | MVPA groups | | | *P*-value ^a^ |
| --- | --- | --- | --- | --- |
|  | No MVPA | Insufficient-MVPA  (<150 minutes/week) | Sufficient-MVPA  (≥150 minutes/week) |  |
| Unweighted n | 393 | 106 | 212 |  |
| Weighted n | 2,618,061 | 774,793 | 1,692,555 |  |
| Weighted % (95% CI) ^b^ | 51.48 (46.7, 56.27) | 15.24 (12.36, 18.11) | 33.28 (28.59, 37.98) |  |
| Years since the first diagnosed with HF | |  |  | .582 |
| <5 years | 37.93 (30.35, 45.52) | 38.77 (27.61, 49.93) | 32.88 (23.27, 42.48) |  |
| ≥5 years | 62.07 (54.48, 69.65) | 61.23 (50.07, 72.39) | 67.12 (57.52, 76.73) |  |
| Age |  |  |  | .429 |
| <65 years | 35.40 (29.08, 41.71) | 42.48 (31.16, 53.8) | 41.37 (30.85, 51.90) |  |
| ≥65 years | 64.60 (58.29, 70.92) | 57.52 (46.20, 68.84) | 58.63 (48.10, 69.15) |  |
| Sex |  |  |  | .015 |
| Male | 43.05 (37.03, 49.07) | 47.31 (35.03, 59.59) | 59.33 (49.39, 69.28) |  |
| Female | 56.95 (50.93, 62.97) | 52.69 (40.41, 64.97) | 40.67 (30.72, 50.61) |  |
| Race/ethnicity |  |  |  | .599 |
| Non–Hispanic white | 69.44 (63.31, 75.56) | 72.1 (63.54, 80.65) | 72.59 (65.6, 79.58) |  |
| Non–Hispanic black | 16.11 (11.71, 20.51) | 18.15 (11.68, 24.62) | 13.00 (8.35, 17.65) |  |
| Mexican American | 4.69 (2.34, 7.04) | 2.29 (0.00, 4.95) | 5.57 (1.70, 9.45) |  |
| Others | 9.76 (6.12, 13.41) | 7.46 (3.63, 11.29) | 8.83 (3.32, 14.35) |  |
| Education level |  |  |  | .029 |
| <High school | 37.26 (32.33, 42.20) | 23.00 (14.28, 31.71) | 24.71 (16.58, 32.84) |  |
| High school | 23.51 (19.02, 27.99) | 31.17 (21.73, 40.62) | 29.25 (21.01, 37.48) |  |
| ≥College or above | 39.23 (33.05, 45.41) | 45.83 (33.60, 58.05) | 46.04 (38.18, 53.91) |  |
| Household income |  |  |  | .080 |
| <$25k | 44.53 (37.19, 51.86) | 38.93 (27.92, 49.94) | 33.84 (25.14, 42.54) |  |
| $25k – <$75k | 47.57 (39.89, 55.25) | 47.15 (32.61, 61.68) | 47.27 (36.75, 57.78) |  |
| ≥$75k | 7.90 (4.26, 11.55) | 13.93 (2.25, 25.60) | 18.89 (11.33, 26.46) |  |
| Marital status |  |  |  | .002 |
| Married or partner | 51.35 (43.67, 59.03) | 53.32 (43.32, 63.32) | 68.31 (60.59, 76.02) |  |
| Smoking status |  |  |  | .326 |
| Currently smoking | 17.14 (12.73, 21.54) | 20.61 (11.62, 29.60) | 23.23 (14.33, 32.12) |  |
| Body mass index |  |  |  | .181 |
| <25 kg/m^2^ | 25.27 (20.42, 30.11) | 20.81 (13.22, 28.39) | 24.72 (15.76, 33.68) |  |
| 25 – 29.9 kg/m^2^ | 20.43 (15.85, 25.00) | 25.23 (13.81, 36.65) | 30.58 (22.93, 38.24) |  |
| ≥30 kg/m^2^ | 54.31 (48.23, 60.39) | 53.97 (41.97, 65.96) | 44.7 (35.62, 53.78) |  |
| Chronic medical conditions (yes) | |  |  |  |
| Coronary heart disease | 37.97 (32.15, 43.78) | 29.18 (17.79, 40.57) | 34.77 (27.57, 41.97) | .379 |
| Cancer | 25.78 (20.39, 31.17) | 27.49 (17.27, 37.71) | 20.97 (13.50, 28.43) | .005 |
| High blood pressure | 81.50 (76.79, 86.21) | 84.14 (76.22, 92.06) | 68.49 (59.5, 77.48) | .861 |
| High cholesterol | 62.99 (57.32, 68.66) | 61.19 (48.78, 73.61) | 64.76 (56.58, 72.94) | .007 |
| Diabetes | 45.55 (38.77, 52.33) | 38.92 (28.19, 49.64) | 28.71 (20.09, 37.33) | .582 |
| Walking difficulty score (Mean; 95% CI) | 1.58 (1.46, 1.70) | 1.26 (1.14, 1.37) | 1.14 (1.06, 1.21) | <.001 |
| HF medications (yes) | 86.70 (82.72, 90.68) | 78.79 (68.46, 89.13) | 80.68 (74.34, 87.02) | .114 |

HF = heart failure; MVPA = moderate- and vigorous-intensity physical activity; NHANES = National Health and Nutrition Examination Survey

Values are the percentages (95% CI) unless otherwise specified (i.e., mean and 95% CI were reported for walking difficulty score). An 8-years combined sampling weight was applied.

^a^ *P*-value is obtained from a Rao-Scott Chi-square test of independence.

^b^ the weighted proportion of the individuals with HF among the US adults

**Online Table 3.**

**Descriptive Characteristics of the Study Population by Sedentary Groups (NHANES 2007 - 2014)**

|  | Sedentary behavior | | *P*-value ^a^ |
| --- | --- | --- | --- |
|  | <8 hours/day | ≥8 hours/day |  |
| Unweighted n | 382 | 329 |  |
| Weighted n | 2,571,343 | 2,514,065 |  |
| Weighted % (95% CI) ^b^ | 50.56 (46.29, 54.84) | 49.44 (45.16, 53.71) |  |
| Years since the first diagnosed with HF | |  | .059 |
| <5 years | 32.67 (26.62, 38.72) | 40.17 (32.19, 48.15) |  |
| ≥5 years | 67.33 (61.28, 73.38) | 59.83 (51.85, 67.81) |  |
| Age |  |  | .906 |
| <65 years | 38.69 (33.34, 44.04) | 38.24 (30.63, 45.84) |  |
| ≥65 years | 61.31 (55.97, 66.66) | 61.76 (54.16, 69.37) |  |
| Sex |  |  | .332 |
| Male | 51.56 (44.90, 58.21) | 46.63 (39.14, 54.11) |  |
| Female | 48.44 (41.79, 55.1) | 53.37 (45.89, 60.86) |  |
| Race/ethnicity |  |  | .444 |
| Non–Hispanic white | 69.02 (62.62, 75.41) | 72.81 (67.14, 78.47) |  |
| Non–Hispanic black | 15.37 (11.27, 19.47) | 15.4 (11.47, 19.34) |  |
| Mexican American | 5.66 (2.42, 8.90) | 3.56 (1.14, 5.97) |  |
| Others | 9.96 (6.65, 13.26) | 8.23 (3.93, 12.54) |  |
| Education level |  |  | .516 |
| <High school | 33.10 (27.29, 38.90) | 28.68 (22.26, 35.09) |  |
| High school | 24.84 (19.43, 30.25) | 28.37 (23.23, 33.52) |  |
| ≥College or above | 42.06 (35.72, 48.41) | 42.95 (35.04, 50.86) |  |
| Household income |  |  | .222 |
| <$25k | 40.98 (34.40, 47.56) | 39.15 (31.52, 46.78) |  |
| $25k – <$75k | 49.33 (42.86, 55.81) | 45.50 (36.72, 54.28) |  |
| ≥$75k | 9.69 (5.43, 13.94) | 15.35 (9.80, 20.89) |  |
| Marital status |  |  | .276 |
| Married or partner | 59.85 (53.65, 66.04) | 54.20 (3.10, 33.96) |  |
| Smoking status |  |  | .300 |
| Currently smoking | 21.92 (15.83, 28.01) | 17.42 (11.26, 23.57) |  |
| Body mass index |  |  | .002 |
| <25 kg/m^2^ | 29.57 (22.97, 36.17) | 19.12 (14.67, 23.58) |  |
| 25 – 29.9 kg/m^2^ | 27.22 (21.88, 32.56) | 21.80 (15.61, 27.98) |  |
| ≥30 kg/m^2^ | 43.21 (37.37, 49.06) | 59.08 (51.42, 66.74) |  |
| Chronic medical conditions (yes) | |  |  |
| Coronary heart disease | 35.12 (29.58, 40.65) | 36.02 (28.78, 43.26) | .844 |
| Cancer | 24.80 (18.45, 31.15) | 24.06 (18.01, 30.12) | .848 |
| High blood pressure | 74.78 (69.21, 80.36) | 80.42 (74.99, 85.85) | .146 |
| High cholesterol | 62.18 (55.92, 68.45) | 64.45 (56.45, 72.44) | .668 |
| Diabetes | 35.04 (28.07, 42.00) | 42.92 (36.53, 49.30) | .094 |
| Walking difficulty score (Mean; 95% CI) | 1.31 (1.23, 1.39) | 1.46 (1.32, 1.61) | .069 |
| HF medications (yes) | 81.11 (75.60, 86.62) | 85.93 (80.73, 91.13) | .204 |

HF = heart failure; NHANES = National Health and Nutrition Examination Survey

Values are the percentages (95% CI) unless otherwise specified. An 8-years combined sampling weight was applied.

^a^ *P*-value is obtained from a Rao-Scott Chi-square test of independence.

^b^ weighted proportion of the individuals with HF among the US adults

**Online Table 4.**

**Joint Associations of MVPA and SB with All-Cause Mortality among Adults with a History of Congestive HF (NHANES 2007–2014)**

| Joint groups | Weighted %  (95% CI) | Hazard ratios (95% CI)  with different reference group ^a^ | | | | | |
| --- | --- | --- | --- | --- | --- | --- | --- |
| SB_(<8 hrs/d)_ + No-MVPA | 20.75  (17.17, 24.33) | **referent** | 1.26  (0.93, 1.72) | 2.21^*^  (1.27, 3.85) | 0.67  (0.45, 0.99) | 1.09  (0.50, 2.38) | 0.96  (0.41, 2.30) |
| SB_(<8 hrs/d)_ + I-MVPA | 7.06  (4.92, 9.19) | 0.72  (0.32, 1.65) | **referent** | 1.60  (0.68, 3.75) | 0.48  (0.22, 1.08) | 0.79  (0.25, 2.54) | 0.70  (0.21, 2.27) |
| SB_(<8 hrs/d)_ + S-MVPA | 22.76  (18.28, 27.23) | 0.45^*^  (0.26, 0.79) | 0.91  (0.70, 1.20) | **referent** | 0.30^*^  (0.17, 0.54) | 0.49  (0.2, 1.21) | 0.44  (0.18, 1.08) |
| SB_(≥8 hrs/d)_ + No-MVPA | 30.73  (26.76, 34.7) | 1.50^*^  (1.01, 2.22) | 2.16^*^  (1.60, 2.90) | 3.32^*^  (1.84, 5.96) | **referent** | 1.64  (0.78, 3.43) | 1.44  (0.63, 3.33) |
| SB_(≥8 hrs/d)_ + I-MVPA | 8.18  (5.81, 10.56) | 0.91  (0.42, 1.98) | 1.26  (0.91, 1.75) | 2.02  (0.82, 4.97) | 0.61  (0.29, 1.28) | **referent** | 0.88  (0.31, 2.47) |
| SB_(≥8 hrs/d)_ + S-MVPA | 10.52  (7.36, 13.69) | 1.04  (0.44, 2.47) | 0.88  (0.62, 1.24) | 2.30  (0.93, 5.70) | 0.69  (0.30, 1.60) | 1.14  (0.40, 3.19) | **referent** |

^a^ Hazard ratio (95% CI) were estimated from the Cox proportional hazard regression model adjusting the study covariates retained using the backward elimination approach (*P*<.20), which include age group, race/ethnicity, education, BMI group, marital status, self-reported medical conditions on coronary heart disease, high cholesterol, diabetes, and walking difficulty score.

^*^ *P* <.05

**Online Table 5.**

**Stratified Associations of MVPA with All-cause Mortality by SB Levels (NHANES 2007 - 2014) ^a^**

|  | Weighted % (95% CI) | Hazard ratio (95% CI) ^b^ | |
| --- | --- | --- | --- |
| SB_(<8 hrs/d)_ |  |  |  |
| No-MVPA | 41.04  (34.73, 47.34) | referent | - |
| I-MVPA | 13.95  (9.62, 18.28) | 0.65  (0.25, 1.66) | referent |
| S-MVPA | 45.01  (37.63, 52.39) | 0.45^*^  (0.25, 0.81) | 0.69  (0.27, 1.76) |
| *P*-for-trend | - | .009 | - |
| SB_(≥8 hrs/d)_ |  |  |  |
| No-MVPA | 62.16  (55.8, 68.53) | referent | - |
| I-MVPA | 16.55  (11.84, 21.25) | 0.53  (0.25, 1.12) | referent |
| S-MVPA | 21.29  (15.41, 27.17) | 0.63  (0.28, 1.42) | 1.19  (0.43, 3.28) |
| *P*-for-trend | - | .259 | - |

^a^ Two Cox proportional hazard regression analyses were conducted by each sedentary group.

^b^ Hazard ratios (95% CI) were estimated from the Cox proportional hazard regression model adjusting the study covariates retained using the backward elimination approach (*P*<.20), which include age group, race/ethnicity, education, BMI group, marital status, self-reported medical conditions on coronary heart disease, high cholesterol, diabetes, and walking difficulty score.

^*^ *P* <.05

**Online Table 6.**

**Stratified Associations of SB with All-cause Mortality by MVPA levels (NHANES 2007 - 2014) ^a^**

|  | Weighted % (95% CI) | Hazard ratio (95% CI) ^b^ |
| --- | --- | --- |
| No-MVPA |  |  |
| SB_(<8 hrs/d)_ | 40.31 (34.65, 45.96) | referent |
| SB_(≥8 hrs/d)_ | 59.69 (54.04, 65.35) | 1.51^*^  (1.01, 2.25) |
| I-MVPA |  |  |
| SB_(<8 hrs/d)_ | 46.31 (34.93, 57.68) | referent |
| SB_(≥8 hrs/d)_ | 53.69 (42.32, 65.07) | 1.16  (0.36, 3.77) |
| S-MVPA |  |  |
| SB_(<8 hrs/d)_ | 68.38 (59.66, 77.09) | referent |
| SB_(≥8 hrs/d)_ | 31.62 (22/91, 40.34) | 2.05  (0.86, 4.87) |

^a^ Three Cox proportional hazard regression analyses were conducted by each MVPA group.

^b^ Hazard ratios (95% CI) were estimated from the Cox proportional hazard regression model adjusting the study covariates retained using the backward elimination approach (*P*<.20), which include age group, race/ethnicity, education, BMI group, marital status, self-reported medical conditions on coronary heart disease, high cholesterol, diabetes, and walking difficulty score.

^*^ *P* <.05

**Online Table 7.**

**Descriptive Characteristics of the US Adults with a History of Congestive HF (NHANES 2007–2018)**

|  | NHANES 2007 – 2018 |
| --- | --- |
| Unweighted n | 1169 |
| Weighted n | 5,450,457 |
| Weighted % (95% CI) ^a^ | 2.41% (2.19 – 2.64) |
| Years since the first diagnose with HF | |
| Mean (95% CI) | 9.13 (8.40 – 9.85) |
| <5 years | 37.58 (33.25 – 41.92) |
| ≥5 years | 62.42 (58.08 – 66.75) |
| Age |  |
| Mean (years) | 66.21 (65.26 – 67.15) |
| <65 years | 37.71 (33.56 – 41.86) |
| ≥65 years | 62.29 (58.14 – 66.44) |
| Sex |  |
| Male | 51.39 (47.96 – 54.81) |
| Female | 48.61 (45.19 – 52.04) |
| Race/ethnicity |  |
| Non–Hispanic white | 68.93 (65.00 – 72.87) |
| Non–Hispanic black | 15.88 (13.03 – 18.72) |
| Mexican American | 4.51 (2.89 – 6.14) |
| Others | 10.68 (8.28 – 13.07) |
| Education level |  |
| <High school | 27.72 (24.23 – 31.21) |
| High school | 28.32 (25.26 – 31.38) |
| ≥College or above | 43.96 (40.36 – 47.56) |
| Household income |  |
| <$25k | 40.66 (37.05 – 44.27) |
| $25k – <$75k | 45.33 (40.69 – 49.97) |
| ≥$75k | 14.00 (10.73 – 17.28) |
| Marital status |  |
| Married or partner | 49.61 (35.25 – 63.96) |
| Smoking status |  |
| Currently smoking | 18.83 (15.53 – 22.13) |
| Body mass index |  |
| <25 kg/m^2^ | 25.02 (21.73 – 28.30) |
| 25 – 29.9 kg/m^2^ | 22.53 (19.67 – 25.39) |
| ≥30 kg/m^2^ | 52.46 (48.48 – 56.43) |
| Chronic medical conditions (yes) |  |
| Coronary heart disease | 39.13 (35.24 – 43.01) |
| Cancer | 23.91 (20.36 – 27.45) |
| High blood pressure | 79.38 (76.16 – 82.60) |
| High cholesterol | 61.60 (57.84 – 65.37) |
| Diabetes | 41.06 (37.32 – 44.79) |
| Walking difficulty score (Mean; 95% CI) | 1.46 (1.39, 1.52) |
| HF medication (yes) | 84.37 (81.61, 87.14) |

HF = heart failure; Values are the weighted percentages (95% CI) unless otherwise specified. A 12-years combined sampling weight was applied.

^a^ weighted proportion of the individuals with HF among the US adults

**Online Table 8.**

**Descriptive Characteristics of the US Adults with a History of Congestive HF by MVPA Levels (NHANES 2007 - 2018)**

|  | MVPA groups | | | *P*-value ^a^ |
| --- | --- | --- | --- | --- |
|  | No-MVPA | Insufficient-MVPA  (<150 minutes/week) | Sufficient-MVPA  (≥150 minutes/week) |  |
| Unweighted n | 618 | 156 | 395 |  |
| Weighted n | 2,694,674 | 731,330 | 2,024,453 |  |
| Weighted % (95% CI) ^b^ | 49.44 (45.75, 53.12) | 13.42 (11.17, 15.67) | 37.14 (33.39, 40.89) |  |
| Years since the first diagnosed with HF | |  |  | .582 |
| <5 years | 41.50 (35.71, 47.28) | 37.64 (28.21, 47.07) | 32.44 (25.35, 39.53) |  |
| ≥5 years | 58.50 (52.72, 64.29) | 62.36 (52.93, 71.79) | 67.56 (60.47, 74.65) |  |
| Age |  |  |  | .429 |
| <65 years | 34.40 (28.86, 39.95) | 41.40 (32.00, 50.81) | 40.66 (33.83, 47.50) |  |
| ≥65 years | 65.60 (60.05, 71.14) | 58.60 (49.19, 68.00) | 59.34 (52.50, 66.18) |  |
| Sex |  |  |  | .015 |
| Male | 44.58 (39.89, 49.28) | 49.5 (39.63, 59.36) | 61.03 (54.48, 67.59) |  |
| Female | 55.42 (50.72, 60.11) | 50.5 (40.64, 60.37) | 38.97 (32.41, 45.52) |  |
| Race/ethnicity |  |  |  | .599 |
| Non–Hispanic white | 66.99 (62.14, 71.83) | 67.73 (59.88, 75.59) | 72.15 (66.98, 77.31) |  |
| Non–Hispanic black | 17.34 (13.6, 21.08) | 19.11 (13.62, 24.60) | 12.69 (9.14, 16.24) |  |
| Mexican American | 4.56 (2.73, 6.40) | 2.75 (0.40, 5.10) | 5.00 (2.72, 7.29) |  |
| Others | 11.11 (7.98, 14.24) | 10.40 (6.16, 14.65) | 10.16 (6.55, 13.78) |  |
| Education level |  |  |  | .029 |
| <High school | 34.11 (29.87, 38.36) | 23.26 (16.47, 30.05) | 20.94 (15.17, 26.72) |  |
| High school | 27.26 (22.78, 31.74) | 27.35 (19.59, 35.12) | 29.91 (23.87, 35.95) |  |
| ≥College or above | 38.62 (33.77, 43.47) | 49.39 (39.66, 59.12) | 49.15 (43.23, 55.07) |  |
| Household income |  |  |  | .080 |
| <$25k | 45.45 (39.69, 51.21) | 40.08 (30.57, 49.59) | 34.67 (28.68, 40.65) |  |
| $25k – <$75k | 44.94 (38.78, 51.10) | 42.64 (31.03, 54.25) | 46.83 (38.85, 54.81) |  |
| ≥$75k | 9.61 (6.05, 13.17) | 17.28 (7.56 – 27.00) | 18.50 (12.57, 24.43) |  |
| Marital status |  |  |  | .002 |
| Married or partner | 49.43 (43.43, 55.44) | 51.42 (42.29, 60.55) | 60.23 (53.02, 67.44) |  |
| Smoking status |  |  |  | .326 |
| Currently smoking | 17.17 (13.48, 20.86) | 22.43 (15.02, 29.85) | 19.60 (13.68, 25.53) |  |
| Body mass index |  |  |  | .181 |
| <25 kg/m^2^ | 27.86 (23.76, 31.97) | 20.10 (13.93, 26.27) | 22.63 (16.12, 29.14) |  |
| 25 – 29.9 kg/m^2^ | 19.34 (15.48, 23.19) | 27.44 (18.51, 36.38) | 25.15 (19.93, 30.37) |  |
| ≥30 kg/m^2^ | 52.80 (47.98, 57.62) | 52.46 (43.00, 61.92) | 52.22 (45.26, 59.19) |  |
| Chronic medical conditions (yes) | |  |  |  |
| Coronary heart disease | 39.51 (34.53, 44.5) | 32.97 (23.43, 42.51) | 40.98 (34.57, 47.39) | .379 |
| Cancer | 25.76 (20.47, 31.05) | 29.77 (20.41, 39.12) | 19.23 (14.21, 24.26) | .005 |
| High blood pressure | 82.20 (78.51, 85.89) | 85.31 (79.01, 91.61) | 73.44 (66.99, 79.89) | .861 |
| High cholesterol | 61.41 (57.02, 65.79) | 59.44 (49.46, 69.41) | 62.99 (57.09, 68.88) | .007 |
| Diabetes | 44.89 (39.5, 50.27) | 36.92 (28.09, 45.74) | 37.17 (30.12, 44.23) | .582 |
| Walking difficulty score (Mean; 95% CI) | 1.66 (1.56, 1.77) | 1.32 (1.22, 1.42) | 1.23 (1.16, 1.30) | <.001 |
| HF medication (yes) | 86.66 (83.68, 89.63) | 80.94 (72.91, 88.97) | 85.57 (77.90, 87.25) | .176 |

HF = heart failure; MVPA = moderate- and vigorous-intensity physical activity; NHANES = National Health and Nutrition Examination Survey

Values are the percentages (95% CI) unless otherwise specified. A 12-years combined sampling weight was applied.

^a^ *P*-value is obtained from a Rao-Scott Chi-square test of independence.

^b^ weighted proportion of the individuals with HF among the US adults

**Online Table 9.**

**Descriptive Characteristics of the Study Population by SB Levels (NHANES 2007–2018)**

|  | Sedentary behavior | | *P*-value ^a^ |
| --- | --- | --- | --- |
|  | <8 hours/day | ≥8 hours/day |  |
| Unweighted n | 639 | 530 |  |
| Weighted n | 2,813,102 | 2,637,355 |  |
| Weighted % (95% CI) ^b^ | 51.61 (47.95, 55.27) | 48.39 (44.73, 52.05) |  |
| Years since the first diagnosed with HF | |  | .016 |
| <5 years | 33.78 (29.05, 38.5) | 41.71 (35.60, 47.82) |  |
| ≥5 years | 66.22 (61.5, 70.95) | 58.29 (52.18, 64.40) |  |
| Age |  |  | .122 |
| <65 years | 40.31 (35.21, 45.42) | 34.84 (29.11, 40.58) |  |
| ≥65 years | 59.69 (54.59, 64.79) | 65.16 (59.42, 70.89) |  |
| Sex |  |  | .225 |
| Male | 53.53 (48.93, 58.14) | 49.03 (43.57, 54.49) |  |
| Female | 46.47 (41.86, 51.07) | 50.97 (45.51, 56.43) |  |
| Race/ethnicity |  |  | .260 |
| Non–Hispanic white | 66.55 (61.39, 71.71) | 71.62 (66.84, 76.40) |  |
| Non–Hispanic black | 16.46 (13.11, 19.81) | 15.20 (11.88, 18.52) |  |
| Mexican American | 4.94 (2.80, 7.08) | 4.00 (2.25, 5.74) |  |
| Others | 12.05 (9.16, 14.94) | 9.18 (5.63, 12.74) |  |
| Education level |  |  | .350 |
| <High school | 30.01 (25.51, 34.52) | 25.37 (20.79, 29.95) |  |
| High school | 26.97 (22.24, 31.70) | 29.63 (25.73, 33.54) |  |
| ≥College or above | 43.02 (37.81, 48.23) | 45.00 (39.18, 50.81) |  |
| Household income |  |  | .141 |
| <$25k | 42.61 (37.61, 47.61) | 38.64 (33.04, 44.25) |  |
| $25k – <$75k | 46.02 (40.42, 51.61) | 44.59 (37.46, 51.71) |  |
| ≥$75k | 11.38 (7.85, 14.91) | 16.77 (11.99, 21.55) |  |
| Marital status |  |  | .241 |
| Married or partner | 55.75 (50.76, 60.73) | 51.54 (45.41, 57.67) |  |
| Smoking status |  |  | .534 |
| Currently smoking | 19.72 (15.65, 23.79) | 17.78 (12.88, 22.68) |  |
| Body mass index |  |  | .034 |
| <25 kg/m^2^ | 27.70 (22.25, 33.14) | 21.87 (17.64, 26.11) |  |
| 25 – 29.9 kg/m^2^ | 24.90 (21.19, 28.61) | 20.11 (15.35, 24.87) |  |
| ≥30 kg/m^2^ | 47.41 (41.78, 53.03) | 58.02 (52.01, 64.03) |  |
| Chronic medical conditions (yes) | |  |  |
| Coronary heart disease | 37.60 (32.59, 42.60) | 40.87 (35.33, 46.41) | .357 |
| Cancer | 23.10 (18.44, 27.76) | 24.70 (19.60, 29.79) | .635 |
| High blood pressure | 79.29 (74.87, 83.72) | 79.44 (74.48, 84.4) | .966 |
| High cholesterol | 63.20 (58.42, 67.98) | 60.16 (54.48, 65.84) | .407 |
| Diabetes | 37.71 (32.09, 43.32) | 44.41 (39.42, 49.41) | .082 |
| Walking difficulty score (Mean; 95% CI) | 1.39 (1.33, 1.45) | 1.53 (1.42, 1.63) | .023 |
| HF medication (yes) | 82.66 (78.77, 86.56) | 86.19 (82.06, 90.33) | .236 |

HF = heart failure; NHANES = National Health and Nutrition Examination Survey

Values are the percentages (95% CI) unless otherwise specified. A 8-year combined sampling weight was applied.

^a^ *P*-value is obtained from a Rao-Scott Chi-square test of independence.

^b^ weighted proportion of the individuals with HF among the US adults

**Online Figure 1.**

**Flow diagram selecting study sample from the NHANES 2007 - 2018**


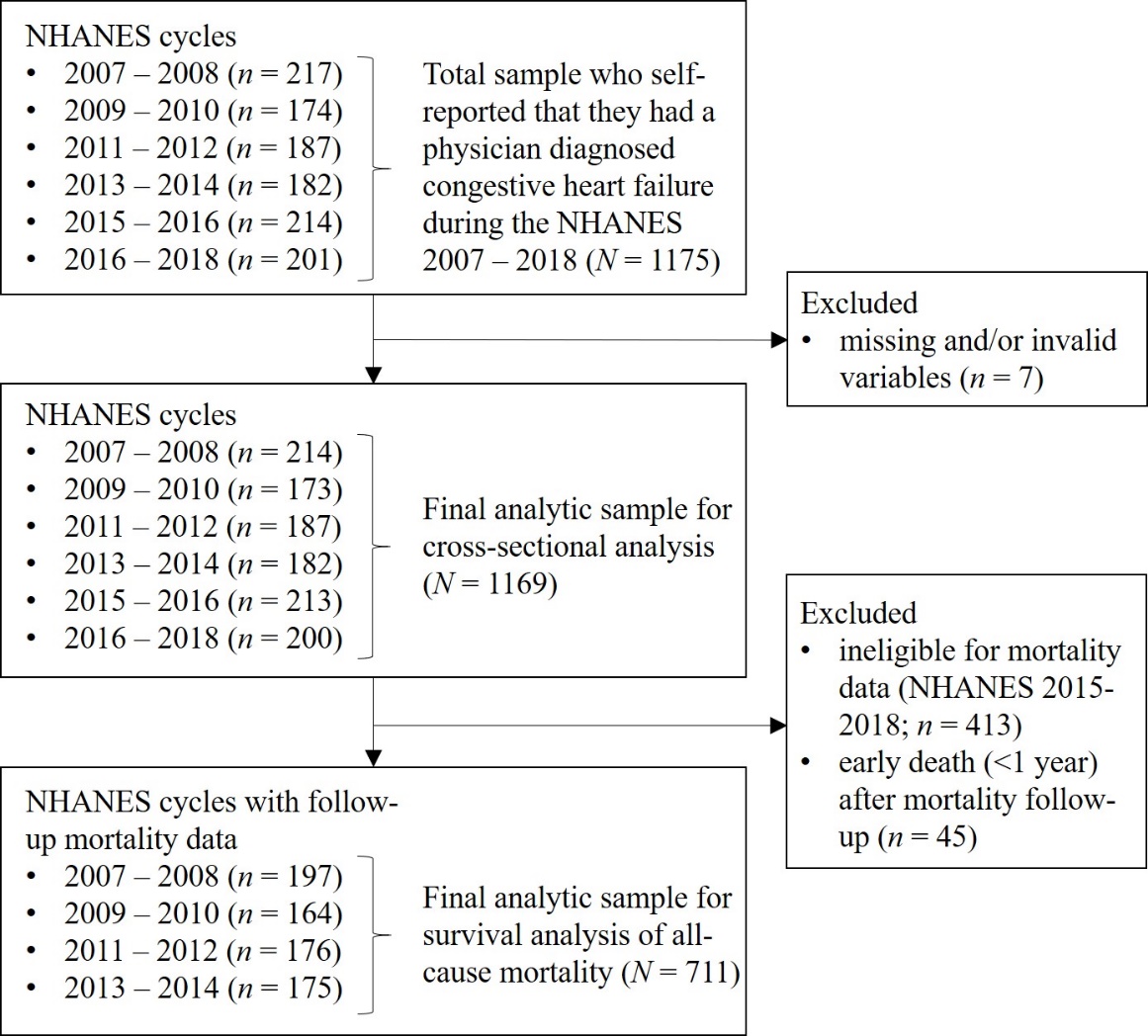


**Online Figure 2.**

Comparisons of the likelihoods of being S-MVPA (reference: No-MVPA + I-MVPA) and SB >8 hours/day (reference: <8 hours/day) between the US adults with and without the history of heart failure (NHANES 2007-2018). The odds ratios were adjusted for study covariates including age, sex, race/ethnicity, education, household income, marital status, smoking status, body mass index, chronic medical conditions, walking difficulty score, and HF medications).


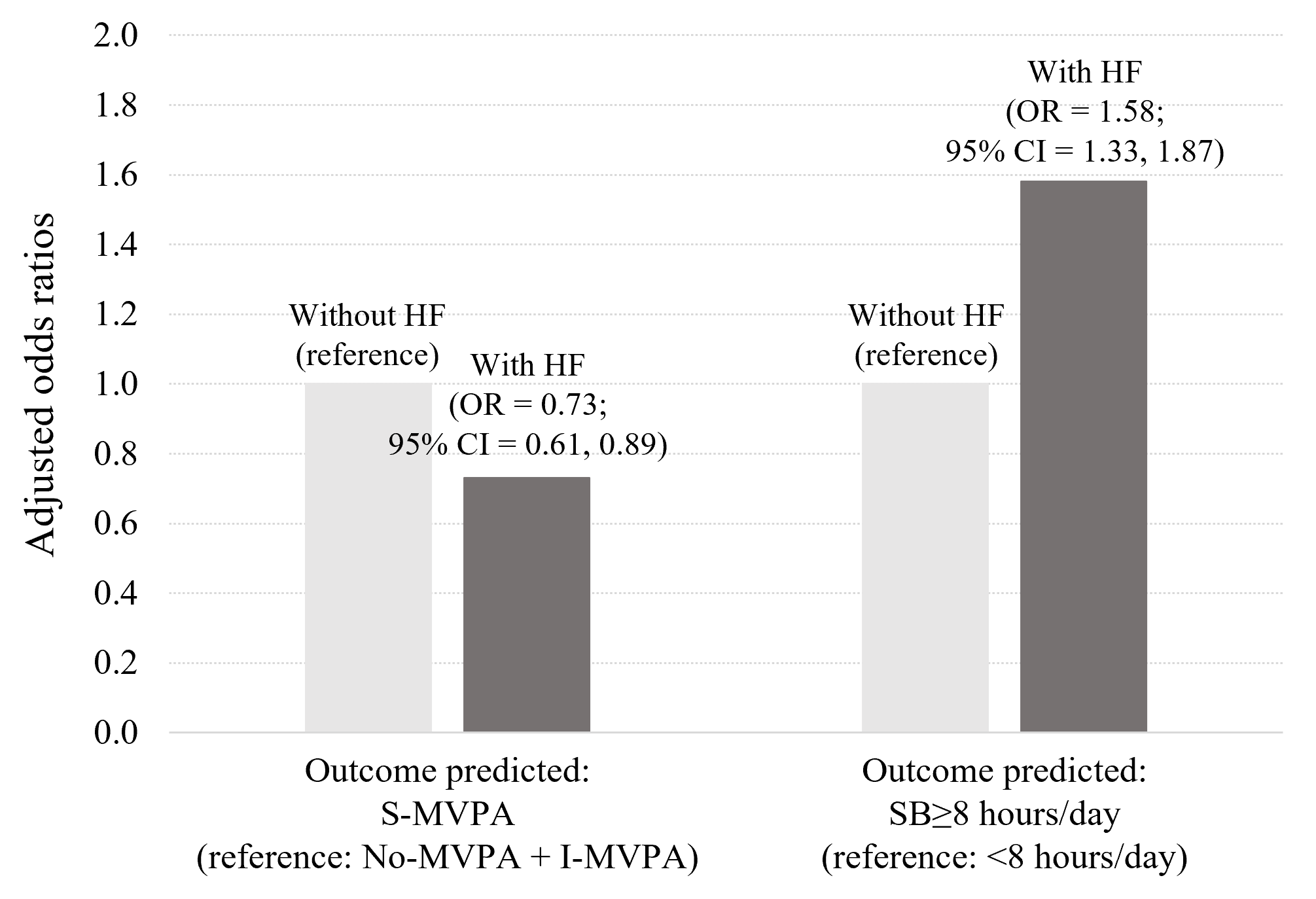

Supplement: S1 File — (DOCX) [file pone.0271238.s001.docx]
